# Weight Management to Reduce Prostate Cancer Risk: A Survey of Men’s Needs and Interests

**DOI:** 10.5539/cco.v5n1p43

**Published:** 2016-05

**Authors:** Amy Schleper, Debra K. Sullivan, J. Brantley Thrasher, Jeffrey M. Holzbeierlein, Jennifer Klemp, Christie Befort, Jill M. Hamilton-Reeves

**Affiliations:** 1 Department of Dietetics & Nutrition, School of Health Professions, University of Kansas Medical Center, 3901 Rainbow Boulevard, Mailstop 4013, Kansas City, Kansas 66160, USA; 2 Department of Urology Surgery, University of Kansas Medical Center, 3901 Rainbow Boulevard, Mail Stop 3016, Kansas City, Kansas 66160, USA; 3 Breast Cancer Prevention Center, University of Kansas Medical Center, 2330 Shawnee Mission Parkway, Mail Stop 3015, Westwood, Kansas 66205, USA; 4 Department of Preventative Medicine and Public Health, University of Kansas Medical Center, 3901 Rainbow Boulevard, Mail Stop 1008, Kansas City, Kansas 66160, USA

**Keywords:** prostate cancer, prevention, diet, weight management, men, technology, health behavior, physical activity, survey, nutrition, awareness, risk

## Abstract

Obese men have a higher rate of prostate cancer-related death than non-obese men, and obesity increases the risk of prostate cancer progression and biochemical recurrence. The purpose of this study was to assess needs and interests of men for a technology-driven weight loss intervention to reduce prostate cancer risk. We distributed a survey collecting demographic characteristics, health history, exercise and eating habits (and perception of those habits), current and prior attempts of health behavior change, and technology use. Survey answers were summarized by count and percent of total respondents. Completed surveys (N = 109) described men with a family history of prostate cancer (25%), a history of elevated prostate specific antigen (26%), and prostate cancer survivors (22%). We compared body mass index (BMI) to perception of weight; overweight and obese men perceived their weight as more normal than their BMI category suggests. Most men reported their diet needed minor improvement (74%), and 65% of men reported they are either currently trying to lose weight or interested in weight loss. Most respondents access the internet (92%), while text messaging (60%) and smartphone application use (40%) are less frequent, especially in men over 60. Our results revealed a need and willingness for lifestyle modification and suggest a need for evidence-based weight loss strategies and for addressing the misperception of weight status. A male-tailored intervention that implements technology could improve energy balance, hold men accountable to healthy behavior change, and promote dietary patterns in order to reduce prostate cancer risk.

## 1. Introduction

Prostate cancer is the most common non-cutaneous cancer in men and the second leading cause of cancer-related mortality in men (Siegel, Miller, & Jemal, 2015). A meta-analysis of 26 studies found that overweight and obesity are responsible for approximately 12-20% of deaths from prostate cancer (Cao & Ma, 2011) For every 5 kg/m^2^ increase in body mass index (BMI), there was a concomitant increase in biochemical recurrence by 21%, and the risk of death from prostate cancer was increased by 15% (Cao & Ma, 2011). Consumption of energy-dense foods (>125 kcal/100g) correlates with an increased risk of highly aggressive prostate cancer (Arab et al., 2013). Weight loss interventions may reduce men’s risk of prostate cancer, but well-designed clinical trials are needed to test this hypothesis. Additionally, since weight management is more commonly studied in women, less is understood about successful lifestyle interventions in men (Pagoto et al., 2012). We know that men are motivated to lose weight for health benefits rather than improved appearance, and they prefer to use physical activity for weight loss rather than using extreme diet limitations (Hankey, Leslie, & Lean, 2002; Lewis et al., 2011). However, more research is needed to evaluate weight loss programs designed around men’s attitudes and interests. In addition to focusing on energy balance, a nutrition intervention focused on prostate cancer should also include an emphasis on diet quality, since qualitative aspects of foods offer further benefits and protection against prostate cancer (Arab et al., 2013).

There is a growing interest in the use of technology for delivering interventions with minimal face-to-face interaction, and web-based technology and smartphone applications have been shown to be effective tools for implementing lifestyle intervention programs (Carter et al., 2013; Fanning, Mullen, & McAuley, 2012; Turner-McGrievy et al., 2013). The purpose of the current study was to assess the needs and interests of men to guide a lifestyle and weight loss intervention to reduce prostate cancer risk. As exploratory objectives, the study collected data on consumption of foods linked to prostate cancer risk, as well as technology use. Given the substantial proportion of men who are overweight and obese and at risk for developing prostate cancer, surveys were distributed to all men to whom prevention or survivorship issues would be relevant.

## 2. Method

### 2.1 Recruitment

We created a survey to collect information about demographic characteristics, health history, exercise habits, perception of eating habits and body weight, prior weight loss attempts, current efforts toward health behavior change, and technology use. Survey answers were summarized by count and percent of total respondents.

We adapted the survey content based on a prior breast cancer survivorship survey conducted at the University of Kansas (KU) Medical Center (Befort, Austin, & Klemp, 2011). Urologic oncologists, dietitians, and psychologists guided survey modifications in order to adapt the survey to men. Consumption of foods linked to prostate cancer risk were surveyed based on the diet and prostate cancer prevention guidelines distributed in the Burns & McDonnell High Risk Prostate Prevention Program (Diggett et al., 2013) and the American Institute for Cancer Research guidelines for prostate cancer risk reduction (“Continuous Update Project Report: Diet, Nutrition, Physical Activity, and Prostate Cancer.,” 2014). Physical activity was assessed using two validated questions from work by Gill and colleagues (Gill et al., 2012).

### 2.2 Informed Consent

The study was approved by the KU Medical Center institutional review board and human subjects’ committee. The team obtained consent without signature (waiver of documentation of consent) from each respondent and provided the subjects with a written statement about the research. The statement informed subjects of the purpose of the research, a basic description of the survey, how their information would be stored, who to contact if they had any questions, and that participation was voluntary. The subjects were offered a copy of the statement for their records.

### 2.3 Survey Distribution

The survey was distributed from September 2013 to November 2013 to members of the Kansas Masonic Foundation and local prostate cancer survivorship support groups. Paper and online versions of the survey were distributed, and surveys were anonymous. Our study sample was derived partly out of convenience, as gatherings related to cancer research and education that included these organizations were held on our campus. Therefore, most participants lived in the Midwestern region of the United States of America. The only inclusion criterion was that the participant be male.

### 2.4 Data Management

In order to protect private health information and restrict access only to the study team, the database and electronic survey were built in Research Electronic Data Capture (REDCap) electronic data capture tools hosted at KU Medical Center (Harris et al., 2009). Counties were classified as metropolitan or nonmetropolitan based on the Rural-Urban Continuum Codes from the 2003 Beale codes (Serhan, Yacoubian, & Yang, 2008). All statistics were performed using count and percent of total respondents using the report features in REDCap and validated with Microsoft® Excel®.

## 3. Results

### 3.1 Survey Response Rate

Of the 68 paper surveys distributed, 57 were returned for a response rate of 84% from paper surveys. Fifty-two surveys were completed online for a response rate of approximately 3% for the electronic survey. Partially completed surveys were included in analysis as each question was analyzed independently. Two surveys were excluded from analysis (one was completed by a woman, and one was a duplicate survey), for a total of 109 surveys analyzed.

### 3.2 Participant Characteristics

Table 1 shows demographic and medical information describing participant characteristics. Twenty-six percent of respondents reported a history of prostate specific antigen elevation, and 25% had a family history of prostate cancer. Twenty-two percent were prostate cancer survivors.

The majority of respondents was White, non-Hispanic men (94%), married (88%), retired (54%) and had at least some college-level education (89%). Eighty-one percent of respondents lived in urban areas (metropolitan), while 19% lived in rural areas (nonmetropolitan).

### 3.3 Weight Status, Perceptions, and Prior Weight Loss

Based on self-reported height and weight, body mass index (BMI) ranged from 20.3 to 54.2 kg/m^2^ (mean BMI 29.5 kg/m^2^ (SD 5.92); median BMI was 28.3 kg/m^2^). Men were asked about self-perception of body weight, and comparisons were made between actual weight and perception of weight. Most men perceived their weight as being “a little overweight” (49%), followed by “about the right weight” (28%), and “too heavy” (22%), but the extent of the respondents’ perceptions did not accurately match the BMI category of their self-reported body weight. Eight percent of obese men and 24% of overweight men reported that they were “about the right weight,” while 41% of obese men and 66% of overweight men reported that they were “a little overweight.” Only 46% of obese men perceived that they were “too heavy.”

As shown in Table 2, 42% of respondents reported that a physician has advised them to lose weight, and 38% reported they are currently trying to lose weight. Among those not currently trying to lose weight (n=60), 48% are interested in weight loss. The majority of men have attempted weight loss in the past (71%), and they used exercise (64%) and/or dieting on their own without assistance (78%) as their weight loss methods. Few other methods were reported, with the next highest method described as commercial programs (9%). Only 6% had used a technology-based weight-loss method (3 men used an internet program, and 3 men used a smartphone application).

### 3.4 Physical Activity

Twenty-nine percent of respondents described their current physical activity level as vigorous, 52% as moderate, or 18% as sedentary (Table 3). Half of the men (50%) considered themselves more active than other men of the same age, and only 10% considered themselves less active than their peers. The most preferred exercise locations included the outdoors (53%), home (51%), or a gym or workout facility (35%).

### 3.5 Diet

Men reported how frequently they consumed various foods. Twenty-six percent of respondents consume red meat daily, and 64% of respondents consume dairy on a daily basis. Eighty-one percent of men consume at least one fruit or vegetable daily. Half of respondents (54%) eat cruciferous vegetables at least once per week, but only 8% consumed cruciferous vegetables on a daily basis. Most men (80%) believe their diets need some improvement and want to make diet changes (Table 3). Spouses are the primary grocery shoppers (54%), followed by the men themselves (30%); many men offered that they equally share this task (15%). Spouses are the primary cooks for 68% of respondents.

### 3.6 Technology Use

Ninety-two percent of the men use the internet, and 66% use the internet daily. Ninety-four percent use email and 74% access their email daily. Home was the highest reported internet use location (91%), followed by phones (34%), and work (23%). Sixty percent of men use short message service (“SMS” or text messaging), 40% use smartphone applications, and only 9% use applications that are health-related (Table 5). When stratified by age, technology use showed a steady decline. Internet and email remain well utilized by men over age 60 (88% and 91%, respectively), however, cell phone or smartphone features are used less in this age group (49% for text messaging and 22% for usage of smartphone applications).

## 4. Discussion

### 4.1 Men’s Interest in Weight Loss

Our survey found men have a high interest in weight loss. A prior study at our facility also found that men at increased risk of prostate cancer are interested in prostate cancer protective lifestyle modifications, and this finding led to the establishment of the Burns & McDonnell High Risk Prostate Prevention Program at KU Medical Center (Diggett et al., 2013). A majority of survey respondents in the current study acknowledged that not only were diet changes needed, they also wanted to make changes—showing potential for future diet intervention. Men who acknowledge a need for improving their diets are more likely to implement diet changes after a prostate cancer diagnosis as a way of empowering themselves to improve their health (Mroz et al., 2010). Additionally, since lifestyle change may be less likely to occur as more time passes after any cancer diagnosis (Lemasters et al., 2014), this supports a need to promote evidence-based lifestyle modifications sooner than later in a man’s life—for prevention of prostate cancer or, should prostate cancer develop, to instigate lifestyle change as soon as possible after diagnosis.

### 4.2 Need for Evidence-Based Programs in Men

Maintaining a healthy weight offers protection against aggressive prostate cancer and prostate cancer mortality (Arab et al., 2013; Bassett et al., 2012; Cao & Ma, 2011), but it is not known if weight loss has an impact on these factors. Men are in need of evidence-based recommendations for weight reduction, and these regimens will be most effective when they cater to men’s needs and interests (i.e., masculinity) (Hunt et al., 2014). Male-specific weight loss regimens should offer straightforward guidelines that provide specific energy restriction targets, providing only limited choice or flexibility to guide their own plan (Coles et al., 2014; Patrick et al., 2011; Young et al., 2012).

A male-tailored intervention should also include exercise as a key component. Sixty-four percent of the men in our study have used exercise as a weight loss method, compared to only 21% of the women in another study using a similar question format (Befort et al., 2011). While not effective alone for weight loss (Jensen et al., 2013) physical activity appeals to men as a weight management strategy, since it emphasizes performance (Sabinsky et al., 2007). Therefore, it could be important for engaging and retaining men in health behavior changes. Physical activity appeals to men with prostate cancer as it is empowering (Lewis et al., 2011). Compared with female cancer survivors, male cancer survivors are approximately 30% more likely to meet the American Cancer Society’s recommendations of >150 minutes of moderate-to-vigorous physical activity per week (Lemasters et al., 2014). Additionally, while data on the benefits of exercise specific to prostate cancer are not conclusive (Young-McCaughan, 2012), men with prostate cancer are more likely to die of cardiovascular disease than the prostate cancer itself (Epstein et al., 2012). Therefore, it is appropriate to include exercise in any lifestyle intervention designed to motivate men and improve prostate cancer-related outcomes.

### 4.3 Diet Patterns and Prostate Cancer Risk

Many foods have been studied for their influence in prostate cancer, and we assessed intake of some of these foods in our survey. Beyond energy targets and exercise, a weight loss intervention aimed at reducing prostate cancer risk should also consider manipulation of these foods. Just over one-fourth of our survey respondents consume red meat daily, and red meat is associated with aggressive prostate cancer risk (Arab et al., 2013; “Food, Nutrition, Physical Activity, and the Prevention of Cancer: a Global Perspective.,” 2007). The majority of respondents consume dairy on a daily basis, and dairy may have a potential role in prostate cancer incidence and aggressiveness (Song et al., 2013). Conversely, a minority of respondents consume cruciferous vegetables daily, which are protective against prostate cancer (Liu et al., 2012). It is important to consider that there is likely an additive and interactive effect of various foods and behaviors. A recent study found that greater adherence to World Cancer Research Fund diet and lifestyle recommendations (maintaining a healthy weight, being physically active, limiting energy-dense foods and beverages, eating more plants and limiting red and processed meats, limiting alcohol intake, and limiting salt intake) resulted in a 13% reduced risk of aggressive prostate cancer for each additional point in the adherence score derived by the researchers (Arab et al., 2013). Therefore, in addition to other lifestyle factors, future interventions targeting weight loss for prostate cancer prevention should also focus on diet quality.

### 4.4 Age and Technology Use

As age increased among our respondents, overall use of technology decreased, but Internet and e-mail remained the more common forms of technology use across all age categories. In our study, 88% of men over age 60 use the internet, compared with 82% of all adults over age 65 nationwide (Smith, 2014). Phones are utilized less, with only 49% of our sample age 60 and older using text messaging and only 22% using smartphone applications in comparison with 35% of all adults over 65 in the U.S. who text (Duggan, 2013). Since application use strongly declines with age, a weight loss intervention using a phone or tablet application may be more difficult to implement in older men, unless these devices and/or technologies are provided to them—only 11% of American adults over age 65 own a smartphone (Duggan, 2013). However, technology-based interventions remain a great option for weight management in men, since men are more drawn to self-guided weight loss compared to group or counselor-driven weight loss (Pagoto et al., 2012). Web-based technology demonstrates an ability to engage and retain men in weight loss interventions (Patrick et al., 2011), and online exercise tracking is more effective in men than in women (Johnson & Wardle, 2011). An application on a smartphone or tablet would further support self-monitoring; a recent study evaluating food tracking tools (n = 128; 77% female) found the highest retention rate with a smartphone application (93%), followed by a website use (55%) and paper diary monitoring (53%). (Carter et al., 2013) Self-monitoring is a critical component for achieving significant weight loss (Burke, Wang, & Sevick, 2011), and it would facilitate the calorie tracking necessary to achieve significant weight loss in men (Young et al., 2012).

### 4.5 Weight Status, Perceptions, and Weight Loss

Consistent with other research, our survey revealed a disconnect between actual weight status and men’s perception of weight status, as our survey respondents demonstrated a tendency to perceive their weight category as lighter than it was. Of those who were clinically obese (BMI ≥ 30), 8% felt they were “about the right weight,” and only 46% felt they were “too heavy.” A similar observation was found in a study of NHANES data from 1999-2006, where 8% of obese men also felt they were about the right weight (Dorsey, Eberhardt, & Ogden, 2009). However, only 24% of the overweight men in our sample (BMI 25.0-29.9) perceived their weight to be the correct weight, which is substantially fewer than the 48% of overweight men from NHANES 2003-2008 data who perceived their weight to be the right weight (Yaemsiri, Slining, & Agarwal, 2011). Prostate cancer-related health education should target these weight misperceptions and emphasize how overweight and obesity not only correlate with more aggressive forms of prostate cancer but also with prostate cancer-related mortality (Haque et al., 2014).

### 4.6 Limitations and Strengths

Surveys are inherently subjective. Height and weight were self-reported, and men, in particular, tend to over report both height and weight (Merrill & Richardson, 2009). Also, a majority of respondents were Caucasian, and 89% had at least some college education, which may limit the generalizability of the findings to men of other races, ethnicities and socioeconomic statuses. Another limitation is that our survey did not assess if the respondents owned smartphones or if they were open to learning and using new technologies, such as smartphone applications. A strength of our study is that the data obtained give us insight into men’s current patterns of technology use, especially by age. We report men’s weight perception, current physical activity, current diet, prior weight loss attempts and patterns of technology, which can better inform the design and tailoring of future intervention trials evaluating the potential role of weight reduction in prostate cancer.

### 4.7 Conclusion

Our survey showed that approximately one-third of the men were currently trying to lose weight, and nearly half of the men not actively attempting weight loss were interested in pursuing weight loss. Many men are interested in weight loss and prefer to use exercise to lose weight (preferably outdoors or at home) and to diet alone without assistance. Future diet education in men should focus on improving intake of protective foods (i.e., cruciferous vegetables), reducing intake of potentially harmful foods (i.e., red meat), and modifying energy intake to improve weight status. Utilizing technology-based delivery may improve adherence, which has potential to improve outcomes. Our data show the majority of men are comfortable using the Internet and e-mail, but smartphone applications or text messaging may fare better in men under the age of 60.

Our results suggest a need and willingness among older men for nutrition interventions targeted at preventing prostate cancer. These data suggest that health care providers may need to address the misperception of weight status. In conclusion, a male-tailored weight management intervention could implement technology to improve energy tracking, hold men accountable to healthy behavior change, and promote dietary patterns in order to reduce prostate cancer risk.

## Figures and Tables

**Table 1:** Demographics and medical history

Participant characteristics (number of respondents)	Mean ± SD or n (%)	Participant characteristics (number of respondents)	n (%)
Age (n=101)	67± 14	Rural vs. Urban (n = 102)	
Race & ethnicity (n=103)		Metro	83 (81%)
White non-Hispanic	97 (94%)	Non-metro	19 (19%)
Hispanic or Latino	0 (0%)	Family history of prostate cancer (n = 103)	26 (25%)
American Indian/Alaska Native	1 (1%)	History of prostate-specific antigen (PSA) elevation (n = 103)	27 (26%)
Black or African American	2 (2%)	Personal history of prostate cancer (n = 101)	22 (22%)
Asian	1 (1%)	Most recent PSA level (n = 102)	
Native Hawaiian /Pacific Islander	1 (1%)	Below or equal to 10 ng/ml	56 (55%)
“Mixed”	1 (1%)	Between 10 ng/ml and 20 ng/ml	4 (4%)
Marital Status (n =104)		Greater than 20 ng/ml	0 (0%)
Single	6 (6%)	Do not know	36 (35%)
Married	92 (88%)	Never been checked	6 (6%)
Divorced/Separated	1 (1%)	How long ago PSA checked (n =100)	
Widowed	5 (5%)	Within past month	12 (12%)
Live alone (n=103)	13 (13%)	Within past 3 months	25 (25%)
Education level (n =103)		Between 6 and 12 months ago	34 (34%)
Less than high school	1 (1%)	More than 1 year ago	20 (20%)
High school	10 (10%)	Never	9 (9%)
Some college	34 (33%)	Employment status (n =102)	
Bachelors	27 (26%)	Full-time (>35 hrs/week)	40 (39%)
Masters	20 (19%)	Part-time (<35 hrs/week)	6 (6%)
Doctoral	11 (11%)	Unemployed	1 (1%)
		Retired	55 (54%)

**Table 2:** Weight status, perceptions, and weight loss

Participant characteristics (number of respondents)	Mean ± SD or n (%)	Participant characteristics (number of respondents)	n (%)
BMI (n = 105)	29.5 ± 5.92	Perception of weight (n = 102)	
Normal weight (BMI 18.5-24.9)	16 (15%)	Too skinny	1 (1%)
Overweight (BMI 25.0-29.9)	50 (48%)	About the right weight	29 (28%)
Obese (BMI ≥ 30.0)	39 (37%)	A little overweight	50 (49%)
Currently trying to lose weight (n = 104)	39 (38%)	Too heavy	22 (22%)
Not currently trying to lose weight, but interested in weight loss (n = 60)	29 (48%)	Weight loss methods (n = 69) ^a^	
Physician advice to lose weight (n = 104)	44 (42%)	Diet on own without assistance	54 (78%)
Prior weight loss (n = 105)		Commercial program	6 (9%)
1-2 attempts	27 (26%)	Diet book or self-help book	5 (7%)
3-4 attempts	25 (24%)	Internet program	3 (4%)
5-6 attempts	4 (4%)	Smartphone Application	3 (4%)
> 6 attempts	19 (18%)	Exercise	44 (64%)
Never tried	30 (29%)	Medications	5 (7%)
Prior weight loss of more than 10 lbs. (n = 60)		Surgery	3 (4%)
1-2 times	30 (50%)	Drinks and/or supplements	2 (3%)
3-4 times	21 (35%)		
5-6 times	4 (7%)		
> 6 times	5 (8%)		

a Respondents could choose more than one weight loss method

**Table 3:** Self-reported physical activity

Participant characteristics (number of respondents)	n (%)
Current PA level (n = 103) ^a^	
Vigorous	30 (29%)
Moderate	54 (52%)
Sedentary	19 (18%)
Perception of PA compared to others same age (n = 104)	
More active	52 (50%)
About as active	42 (40%)
Less active	10 (10%)
PA location (n=104) ^b^	
Home	53 (51%)
Gym, community center, etc.	36 (35%)
Outdoors	55 (53%)
No intentional PA	6 (6%)
Other	10 (10%)
Occupational PA (n=103)	
Heavy labor/manual work	6 (6%)
Walking with some light lifting	31 (30%)
Sitting/standing with some walking	28 (27%)
Mainly sitting	19 (18%)
Other	19 (18%)

a Survey question adapted from Gill, D. P., G. R. Jones, G. Zou, and M. Speechley. 2012. Using a single question to assess physical activity in older adults: a reliability and validity study. *BMC Medical Research Methodology* 12:20. doi:10.1186/1471-2288-12-20.

b Respondents could choose more than one PA location.

**Table 4:** Self-reported diet

Participant characteristics (number of respondents)	n (%)
Primary grocery shopper (n = 104)	
Self	31 (30%)
Spouse	56 (54%)
Caregiver	1 (1%)
Self & spouse (both)	16 (15%)
Primary cook (n = 105)	
Self	27 (26%)
Spouse	71 (68%)
Caregiver	1 (1%)
Eat out most of the time	2 (2%)
Self & spouse (both)	4 (4%)
Perception of current diet (n = 103)	
Healthy, with no change needed	18 (17%)
Healthy, with minor change needed	76 (74%)
Needs change, and want to change	6 (6%)
Not healthy, and do not want to change	3 (3%)

**Table 5:** Self-reported technology use

Participant characteristics (number of respondents)	n (%)
Internet frequency (browse/surf; n = 108)	
Daily	71 (66%)
1-3×/week	20 (19%)
1-3×/month	2 (2%)
Rarely	6 (6%)
Do not use	9 (8%)
Internet use location (n = 100) ^a^	
Home	91 (91%)
Phone	34 (34%)
Work/office	23 (23%)
iPad	1 (1%)
E-mail use (n = 108)	
Daily	80 (74%)
1-3×/week	14 (13%)
1-3×/month	1 (1%)
Rarely	6 (6%)
Do not use	7 (6%)
Text messaging frequency (n = 108)	
Daily	27 (25%)
1-3×/week	20 (19%)
1-3×/month	5 (5%)
Rarely	13 (12%)
Do not use	43 (40%)
Smartphone Application use (n = 108)	
Yes	43 (40%)
No	65 (60%)
Health-related smartphone application	10 (9%)

a Respondents could choose more than one location of internet use
